# Bmi1 Severs as a Potential Tumor-Initiating Cell Marker and Therapeutic Target in Esophageal Squamous Cell Carcinoma

**DOI:** 10.1155/2020/8877577

**Published:** 2020-08-20

**Authors:** Xiaochen Wang, Kang Li, Maosheng Cheng, Ganping Wang, Hui Han, Fangfang Chen, Wenjing Liao, Zhi Chen, Jianwen Chen, Yong Bao, Liang Peng, Demeng Chen

**Affiliations:** ^1^Center for Translational Medicine, Institute of Precision Medicine, The First Affiliated Hospital, Sun Yat-sen University, Guangzhou 510030, China; ^2^Department of Radiation Oncology, The First Affiliated Hospital of Sun Yat-sen University, Guangzhou 510030, China; ^3^Oncology Department, Chinese PLA General Hospital, Beijing 100000, China

## Abstract

Esophageal squamous cell carcinoma (ESCC) is a frequent malignant tumor with low 5-year overall survival. Targeting ESCC tumor-initiating cells (TICs) may provide a new research avenue to achieve better therapeutic effects of ESCC. However, the identity and characteristics of ESCC TICs remain poorly understood. Through genetic lineage tracing approach, we found that a group of Moloney murine leukemia virus insertion site 1- (Bmi1-) expressing cell populations present in the invasive front of the esophageal epithelium, providing a continuous flow of tumor cells for ESCC. Subsequently, we found that ablation of Bmi1^+^ cells from mice with ESCC led to inhibition of tumor growth. In addition, our results demonstrated that PTC-209, an inhibitor of Bmi1, was able to inhibit ESCC progression when combined with cisplatin. In summary, our data suggest that Bmi1^+^ cells serve as TICs in ESCC.

## 1. Introduction

Esophageal cancer is one of the most commonly diagnosed cancers, ranking the sixth cancer-related mortality worldwide [1]. It is mainly composed of two histological types: esophageal squamous cell carcinoma (ESCC) and esophageal adenocarcinoma (EAC). ESCC accounts for more than 90% of the whole esophageal cancer cases in China [2]. However, early diagnosis of ESCC is hard to achieve, resulting in a majority of the ESCC patients diagnosed at advanced stages. And the five-year survival rate of ESCC patients remained around 10% owing to high recurrence and distant metastasis [3]. Recently, studies have shown that tumor-initiating cells (TICs) or cancer stem cells are the main cause of tumor recurrence and metastasis [4, 5]. Hence, understanding TICs in ESCC might provide some novel insights in how to improve current treatment of ESCC.

TICs are the cells that are able to self-renew and evolve into the heterogeneous lineages of cancer cells that make up the tumor population [6]. TICs were first identified in leukemia, which have shown that only a limited proportion of transplanted primary tumor cells could cause secondary tumors [7]. Since then, TICs have been successfully isolated from multiple solid tumors by fluorescence-activated cell sorting (FACS) and cell surface markers [8–10]. In ESCC, CD44 is the major marker used in isolation and detection of TICs. For example, CD44^+^/CD24^−^ ESCC cells exhibited higher oncogenous potential in vivo [11]. In addition, selecting cells using both CD44 and ALDH can increase the enrichment effect of TICs by more than 10 times [12]. Other surface proteins, such as integrin *α*7, CD90, and Cripto-1, have been reported as potential TIC markers in ESCC [13–15]. However, traditional transplantation assays often disrupt the native microenvironment of TICs and led to alteration of TIC characteristics [16]. To avoid the shortcomings of these assays, lineage tracing of genetically labeled cells has been used to identify TICs in vivo [17–19].

Moloney murine leukemia virus insertion site 1 (Bmi1) is the core component of the polycomb repressive complex 1 (PRC1), which mediates gene silencing through monoubiquitination of histone H2A [20, 21]. Bmi1 functions as a significant stem cell self-renewal factor. It is also involved in multiple tumorigenic processes, including cell migration, invasion, epithelial-mesenchymal transition (EMT), and chemotherapy resistance [22, 23]. The expression of Bmi1 is associated with the progression and invasion of ESCC [24]. Moreover, it is a potential biomarker for the early diagnosis of ESCC [25]. Importantly, the expression of Bmi1 in p75^NTR^-positive ESCC TICs was higher than that in p75^NTR^-negative cells [26], which indicates the stemness feature of Bmi1. Study has shown that downregulation of CD44 and Bmi1 in ESCC TICs by administration of nontoxic AUR improved the effect of chemotherapy [27]. Based on these researches, we speculate that Bmi1^+^ tumor cells might mark CSCs and provide a novel therapeutic molecular target in ESCC.

This article shows that Bmi1^+^ cells can represent TICs in ESCC and analyzes the related therapeutic value. For this purpose, we used a mature genetic lineage tracing technique, in which the mice with ESCC were induced by 4-nitroquinoline (4NQO). We found that (1) the gene ablation of Bmi1 led to increased apoptosis, decreased proliferation, and weakened stemness of ESCC; (2) the Bmi1^+^ tumor cells led to the progressive growth of epithelial clones and the Bmi1^+^ tumor cells were tumor-initiating cells in ESCC; and (3) the cisplatin combined with Bmi1 targeting drug could effectively inhibit tumor growth in ESCC.

## 2. Materials and Methods

### 2.1. Animal Assays

All animal use protocols and experiments have been approved by the Institutional Animal Care and Use Committee (IACUC), Sun Yat-sen University. The approval number is SYSU-IACUC-2019-000077. All the animal experiments were carried out in the Laboratory Animal Center, Sun Yat-sen University.

*Bmi1CreER*, *Rosa^tdTomato^* and *Rosa^DTA^* mice were obtained from The Jackson Laboratory. To induce ESCC formation in mice, 4NQO (Sigma, N8141) was prepared with 1,2-propanediol (Sigma, 8223245000) into a 10 mg/mL stock solution and diluted with water for 20 times. The mice aged 8 weeks were fed for 16 weeks and drank normal water for 4 weeks. For lineage tracing, after 16 weeks of 4NQO treatment, in *Bmi1CreER;Rosa^tdTomato^* or *Bmi1CreER;Rosa^tdTomato^;Rosa^DTA^* transgenic mice, tamoxifen (0.08 mg/g body weight per day for 3 days; Sigma, T5648-1G) was injected intraperitoneally. Esophageal samples were collected at different time points and frozen sectioned. The expression and distribution of tdTomato fluorescence protein were analyzed under a fluorescence microscope. For drug treatment assay, Bmi1CreER;Rosa^tdTomato^ mice with ESCC were divided into four different treatment groups, including control group, cisplatin (1 mg/g mouse weight every week; Sigma, BP809) treatment group, PTC-209 (1 mg/g mouse weight every week; MCE, HY-15888-5 mg) treatment group, and cisplatin (0.5 mg/g mouse weight every week)+PTC-209 (0.5 mg/g mouse weight every week) treatment group. After 4 weeks of drug treatment, ESCC samples were collected and analyzed.

### 2.2. Hematoxylin and Eosin Staining

The specimens of ESCC with the adjacent mucosal tissues were fixed in 4% paraformaldehyde (biosharp, BL539A) for up to 24 h and embedded in paraffin. Then, the section with a thickness of 5 *μ*m was cut from the paraffin block and stained with hematoxylin eosin (H&E) kit following the manufacturer's instruction (Solarbio, G1120-100). Briefly, after heating at 65°C, the paraffin sections were dewaxed in xylene and hydrated by serial washing in graded ethanol and distilled water, followed by staining in hematoxylin for 5 minutes, color separation with 1% hydrochloric acid alcohol for 5 seconds, and staining in eosin for 2 minutes. Then, the paraffin sections were dehydrated with graded series of ethanol, removed with xylene, and finally sealed with neutral balsam (Macklin, 822941-100 g).

### 2.3. Immunohistochemistry (IHC) Staining

Dewaxed paraffin sections were obtained by the same method as mentioned above. Sections were treated with heat-induced epitope recovery with sodium citrate buffer (Bioss, C02-02002), followed with blocking endogenous peroxidase prior to primary antibody incubation. Specific primary antibodies including CD44 (1 : 200; Abcam, ab157107), Tp63 (1 : 200; Abcam, ab53039), and KRT5 (1 : 200; Abcam, ab52635) were used. Horseradish peroxidase (HRP) conjugate was used for DAB staining. Expression levels were based on staining intensity and area of tumor cells.

### 2.4. Flow Cytometric Sorting

Cancer cells were isolated from the esophagus of tamoxifen-induced *Bmi1CreER;Rosa^tdTomato^* mice through combining mechanical dissociation with enzymatic degradation by Tumor Dissociation kits (Miltenyi, 130-096-730). To be specific, 0.04-1 g tumor tissue was cut into small pieces of 2-4 mm^3^ and was dissociated in a volume of approximately 2.5 mL enzyme mix. Then, isolated cells were filtered through a 75 *μ*m diameter mesh and centrifuged at 300 × g for 7 minutes and supernatant was aspirated completely. Then, cells were washed with 1 × PBS and resuspended with 90 *μ*L 1 × PBS per 10^7^ total cells. Then, anti-EpCAM (1 : 500; Abcam, ab221552) was added and reaction solution was incubated for 30 minutes on ice. Cells were washed and resuspended in 500 *μ*L 1 × PBS and incubated with a secondary antibody (1 : 1000; Abcam, ab6717) for 25 minutes on ice. Then, cell sorting was performed on BD FACSAria II. EpCAM^+^Tomato^+^ cells were collected. All steps were performed under sterile conditions.

### 2.5. Tumorsphere Formation Assays

Cells were seeded in 6-well ultralow attachment plates at 3,000 cells/mL in stem cell medium as previously described [18]. Cells were cultured in a humidified 5% CO_2_ incubator at 37°C for 7-10 days, during which serum-free media were changed every other day until the spheres formed. Then, tumorspheres were collected, washed with 1 × PBS, and incubated with Trypsin-EDTA for two minutes at 37°C. Then, the number of tumorspheres was counted. Three dishes were used for each group and all experiments were repeated three times.

### 2.6. TUNEL Assays

TUNEL assays are carried out using a commercial kit (KeyGEN, KGA703). According to the instructions provided by the manufacturer, freezing sections were fixed in 4% paraformaldehyde fix solution at room temperature (15-25°C) for 20-30 minutes and rinsed in 1 × PBS three times for 15 minutes. Then, sections were treated with 100 *μ*L proteinase K for 30 minutes at 37°C and rinsed in 1 × PBS three times for 15 minutes again. After being immersed in 3% H_2_O_2_ sealing liquid for 10 minutes and rinsed, the slides were added with 100 *μ*L DNase I reaction liquid containing 2000-3000 U, 40-60 *μ*L DNase I (50 U/*μ*L), and 60-40 *μ*L DNase I buffer and rinsed. Later, TdT enzyme reaction solution, streptavidin-HRP working fluid, and DAB working fluid were added 50 *μ*L after each time the slides were rinsed in 1 × PBS three times for 15 minutes and drained with blotting paper. Dyed in hematoxylin stain for 30 seconds to 5minutes and washed with distilled water, the slides were put in methanol hydrochloride solution for differentiation for 5 seconds and washed with distilled water again, followed by 70%, 85%, 95%, and absolute ethyl alcohol each for 5-minute rinse and xylene twice for ten minutes. After being dried, the samples were added with neutral balsam (Macklin, 822941-100 g), covered with glass slides, photographed, and observed under an optical microscope.

### 2.7. In Situ Hybridization

Dewaxed paraffin sections were obtained by the same method as H&E. Incubation with 0.3% H_2_O_2_ at room temperature for 30 minutes was done to remove endogenous peroxidase activity. After washing with ddH_2_O, proteinase K was dripped onto paraffin sections and incubated at 37°C for 25 minutes. Then, the probe-free hybridization solution was added to pre-treat the slice (this step can be omitted). After washing with ddH_2_O, the probe-containing hybridization solution was added and incubated at 46°C overnight. Sections were soaked three times with 46°C ddH_2_O for 15 minutes each. Peroxidase-labeled sheep anti-digoxin antibody was added and incubated at room temperature for 2 hours. Observation under an optical microscope after color development with DAB was done.

### 2.8. Statistical Analysis

The two-tailed unpaired Student *t*-test was used to measure statistical significance. Data were presented as mean ± SD. All the data statistical analysis is carried out through GraphPad Prism 8.0. For an alpha probability of 0.05, the sample size needed to detect statistical significance difference is at least 6 animals in each group.

## 3. Results

### 3.1. Expression Levels and Clinical Value of Bmi1 in Esophageal Carcinoma

**Figure 1:**
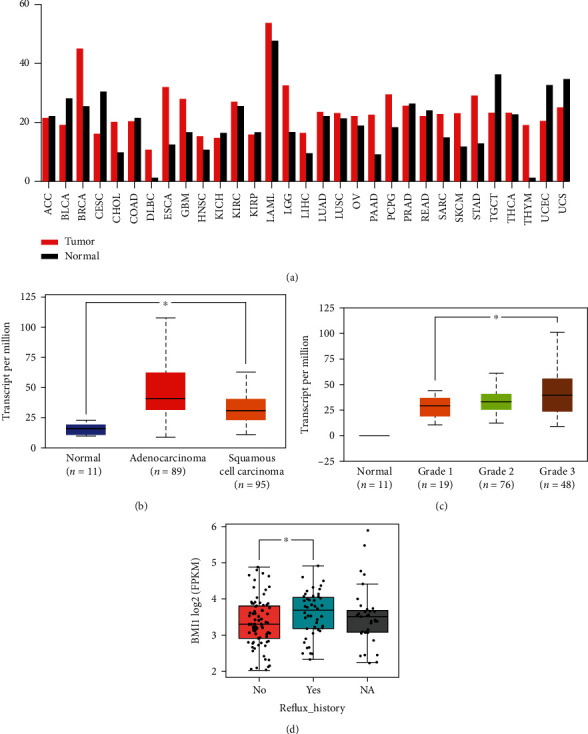
High expression of Bmi1 in esophageal carcinoma. (a) The TCGA database demonstrated that Bmi1 gene expression was significantly upregulated in esophageal carcinoma tissues. (b) Bmi1 expression levels in ESCA based on tumor histology were compared with normal tissues in the TCGA database (*n* = 195). (c) Bmi1 expression levels in different grades of esophageal carcinoma from TCGA database. (d) Gastroesophageal reflux is more likely to occur in patients with high expression of Bmi1. No = patients without gastroesophageal reflux; Yes = patients with gastroesophageal reflux; NA = data missing. ^∗^*p* < 0.05.

According to the data of The Cancer Genome Atlas (TCGA) database, the expression level of Bmi1 mRNA in esophageal carcinoma (ESCA) was upregulated in the esophageal carcinoma tissues compared with normal tissues (Figure 1(a)). And both ESCC and EAC tissues' Bmi1expression levels were significantly higher than normal tissues (Figure 1(b)). In addition, the expression of Bmi1 was correlated with the tumor grade of ESCA; although there were marginal differences between grades 1 and 2 and grades 2 and 3, there was still significant difference between grades 1 and 3 (Figure 1(c)). Furthermore, the clinical phenotype of Bmi1 expression pattern demonstrated that patients with gastroesophageal reflux tended to express higher Bmi1 leve1 (Figure 1(d)). And gastroesophageal reflux was considered to be one of the precancerous diseases of esophageal cancer [28]. Overall, these data indicated the potential clinical significance of Bmi1 in esophageal cancer patients.

### 3.2. Bmi1^+^ Cells Are the TICs in Mouse ESCC

**Figure 2:**
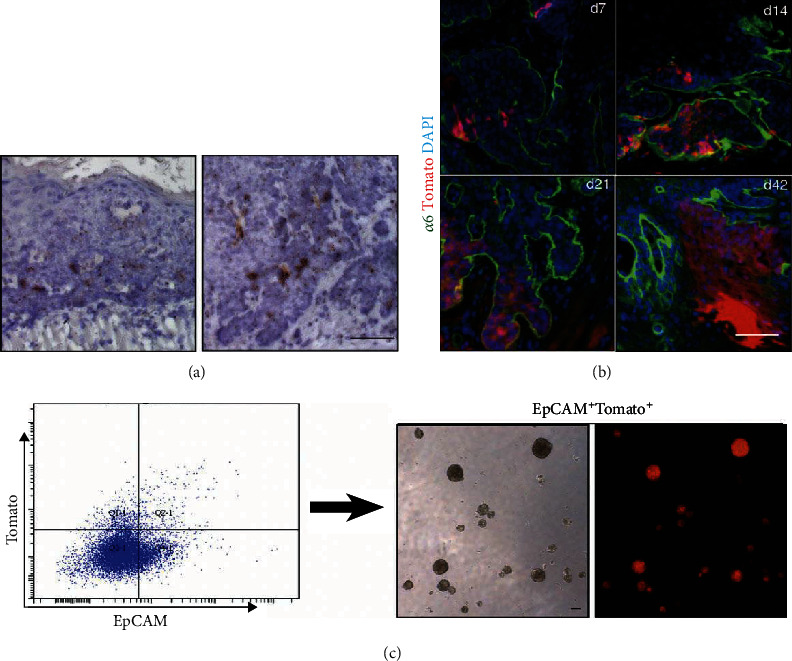
Bmi1^+^ cells and their progeny cells can self-renew in vivo and in vitro. (a) In situ hybridization of Bmi1 in ESCC samples induced by 4NQO for 15 weeks (left) and 22 weeks (right). The brown area is the expression signal of the Bmi1 gene. (b) Bmi1^+^ cells are labeled in *Bmi1CreER;Rosa^tdTomato^* mice following a single dose of tamoxifen and traced for 7, 14, 21, and 42 days. Red represents Tomato-positive Bmi1-expressing cells and their differentiated progeny cells. Green labels integrin *α*6, a marker of the epithelial basement membrane. Nuclei are labeled in blue. (c) Three days after tamoxifen injection, EpCAM^+^Tomato^+^ double-positive cells sorted by flow cytometry were cultured into tumorspheres in vitro. Scale bars are 100 *μ*m.

To investigate whether Bmi1 is expressed in the mouse ESCC, we performed in situ hybridization assay. Our results indicate that Bmi1 is expressed in the subsets of ESCC cells in the areas near the basement membrane 15 weeks and 22 weeks after 4NQO treatment (Figure 2(a)), suggesting a possible role of Bmi1-expressing cells. To verify whether Bmi1-expressing cells have a role in ESCC, we bred *Bmi1CreER;Rosa^tdTomato^* transgenic mice and carried out lineage tracing assay. Our data showed that a small number of Tomato^+^ cells were presented near the basement membrane 7 days after tamoxifen injection. In the samples taken at 14 days, 21 days, and 42 days, the number of Tomato^+^ cells increased with time and distributed in both the epithelial basal layer and the epithelial interstitial layer. Especially on the 42nd day, Tomato^+^ cells were widely distributed in the esophageal epithelium (Figure 2(b)). This shows that in ESCC, the offspring cells differentiated from Bmi1^+^ cells located in the basement membrane gradually develop into tumor parenchyma cells of ESCC over time. To further probe the stemness of Bmi1^+^ cells, we harvested ESCC mice three days after injection of tamoxifen and isolated EpCAM^+^Tomato^+^ double-positive cells for sphere formation assay. We found that ESCC Bmi1^+^ cells could form spheres in vitro (Figure 2(c)). Together, these data showed that the Bmi1^+^ cells served as TICs in mouse ESCC.

### 3.3. Bmi1^+^ Cells Are Critical for ESCC Progression

**Figure 3:**
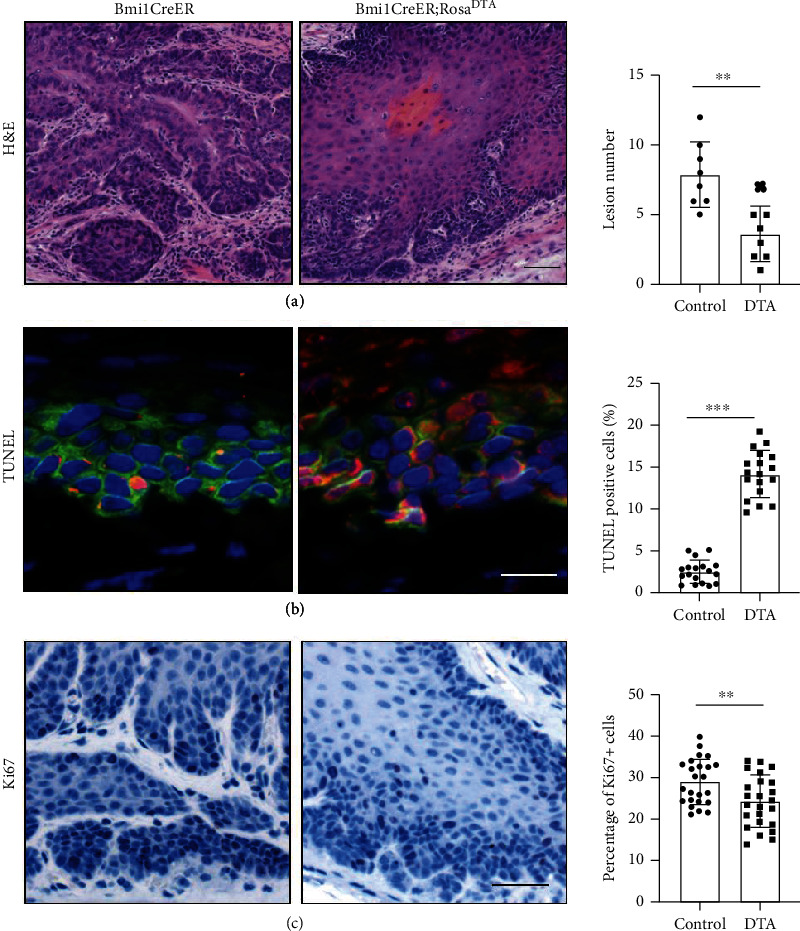
Depletion of Bmi1^+^ cells led to decreased proliferation and increased apoptotic phenotype in ESCC. (a) *Bmi1CreER;Rosa^tdTomato^;Rosa^DTA^* mice were compared with the control in the malignant degree of ESCC. (b) Apoptosis of the *Bmi1CreER;Rosa^tdTomato^;Rosa^DTA^* group was higher than that of the control group under the TUNEL test. Red indicates apoptotic cells. (c) Ki67 signal was reduced in ESCC without Bmi1^+^ cells, and Ki67 signal was represented by black. ^∗^*p* < 0.05, ^∗∗^*p* < 0.01, and ^∗∗∗^*p* < 0.001. Scale bars are 100 *μ*m.

In order to explore the role of Bmi1^+^ cells in the progression of ESCC, we also treated *Bmi1CreER;Rosa^tdTomato^; Rosa^DTA^* mice with 4NQO. At 16 weeks of 4NQO treatment, Bmi1^+^ cells underwent apoptosis caused by diphtheria toxin after tamoxifen injection, resulting in almost no Bmi1^+^ cells in the tumor. After the removal of Bmi1^+^ cells, we can see that the lesion number was reduced (Figure 3(a)). Our results also showed that after the removal of Bmi1^+^ cells, the proportion of apoptosis of ESCC cells increased and the ability of proliferation was decreased (Figures 3(b) and 3(c)). These results indicate that Bmi1^+^ cells are important for the growth of ESCC.

### 3.4. The Expression of ESCC TIC Markers Was Inhibited after Bmi1^+^ Cell Ablation

**Figure 4:**
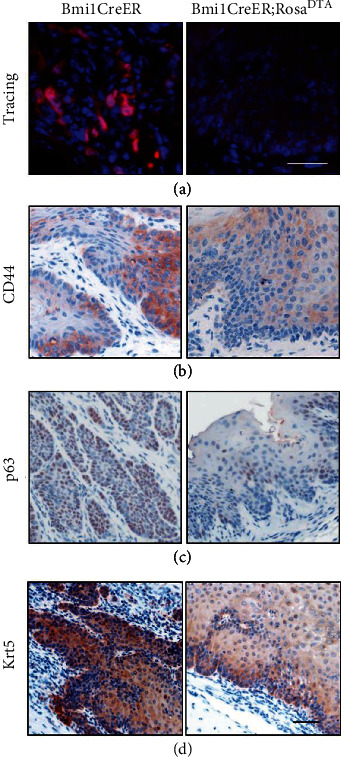
The stemness phenotype of ESCC without Bmi1^+^ cells was inhibited. (a) Samples of control and *Bmi1CreER;Rosa^tdTomato^;Rosa^DTA^* mice were collected on the 21st day after a single intraperitoneal injection of tamoxifen. Bmi1^+^ cells of *Bmi1CreER;Rosa^tdTomato^;Rosa^DTA^* mice were effectively removed. Red labels Bmi1^+^ cells and their progenies. (b–d) In ESCC samples, all three types of TIC markers, CD44 (b), tp63 (c), and Krt5 (d), were reduced after Bmi1^+^ cells were removed. Brown represents the signals of interest. Scale bars are 100 *μ*m.

Further, we examined the expression of known ESCC TIC markers, CD44, tp63, and KRT5, in ESCC tissue after Bmi1^+^ cell removal. Our data showed that Bmi1^+^ cells in ESCC were almost eliminated after tamoxifen administration (Figure 4(a)). In the absence of Bmi1^+^ cells, we found that the expression of three tumor stem cell markers (CD44, Tp63, and Krt5) decreased in ESCC (Figures 4(b)–4(d)). It shows that after the scarcity of Bmi1^+^ cells, the stemness characteristics of ESCC are largely inhibited, suggesting that Bmi1 is important for the stemness of ESCC.

### 3.5. Cisplatin Combined with PTC-209 in the Treatment of ESCC

**Figure 5:**
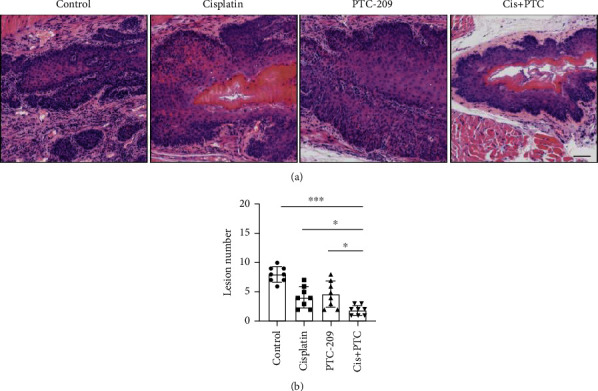
Treatment effect of cisplatin combined with PTC-209 on ESCC. (a, b) From left to right, the histopathology (a) and histogram (b) of ESCC in the control group, cisplatin-treated group, PTC-209-treated group, and the cisplatin^+^PTC-209-treated group. The processing time of the four groups was four weeks. Cisplatin combined with PTC-209 has the lowest malignancy. ^∗^*p* < 0.05, ^∗∗^*p* < 0.01, and ^∗∗∗^*p* < 0.001. Scale bars are 100 *μ*m.

Our previous studies have proved that Bmi1 is an important tumor stem cell marker of ESCC, so the targeted therapy of Bmi1^+^ cells may provide a possibility for the clinical treatment of ESCC. Based on the above data, we carried out the animal drug experiment of cisplatin combined with PTC-209 (a small molecular inhibitor of Bmi1). The experimental results show that although cisplatin and PTC-209 alone have a certain therapeutic effect, the combination of cisplatin and PTC-209 has the least lesion number and the best therapeutic effect (Figures 5(a) and 5(b)).

## 4. Discussion

Bmi1 plays the important part in maintaining the dynamic balance of mitochondrial function and redox and can function like stem cells and progenitor cells to regulate cellular metabolism [29]. Notably, despite the key role of Bmi1 in the self-renewal of various somatic cancer stem cells that has been reported [30], there is no research focusing on Bmi1 in ESCC. In our study, we first confirmed the high expression of Bmi1 in esophageal carcinoma and patients with high expression prone to malignant transformation. After that, we generated *Bmi1CreER;Rosa^tdTomato^* transgenic mice and explored the role of Bmi1^+^ cells as a source of carcinogenesis in ESCC. We utilized genetic lineage tracing analysis [31] and identified a subpopulation of Bmi1^+^ tumor cells that give rise to progressively growing epithelial clones. In addition, we demonstrated that genetic ablation of Bmi1 resulted in increased apoptosis and decreased proliferation. We also compared different treatment strategies of ESCC, and we found that the therapeutic effect of targeting both the tumor bulk and TICs was better than monotherapy [32–34].

The esophagus has a keratinized squamous epithelium consisting of four to five cell layers with rapid turnover characteristics, which is maintained by proliferative basal cells. These basal cells can renew, differentiate, and migrate to the lumen, producing the upper basal layer of terminally differentiated cells. In previous studies, clonal analysis using CreER transgenic mice suggests that Sox2- and K15-labeled progenitor and/or stem cell populations have a higher potential for self-renewal than that of committed progenitors [35, 36]. In the present study, Bmi1 actively expressed in the ESCC and Bmi1^+^ cells were enriched in ESCC with stem cell properties. Importantly, Bmi1^+^ cells differentiated into a large chunk of the tumor of the *Bmi1CreER;Rosa^tdTomato^* mouse over time, consistent with previous studies [37, 38]. Moreover, Bmi1^+^ cell depletion by using Bmi1CreER;Rosa^tdTomato^;Rosa^DTA^ mouse further proves that Bmi1^+^ cells function as TICs in ESCC.

In terms of the treatment of esophageal cancer, the effect of monotherapy is not all satisfactory. Cisplatin is capable of killing proliferative cells [39] but has limited effect on relatively resting cells such as TICs, resulting in poor efficacy. Our study showed that Bmi1^+^ cells are endowed with malignant phenotype. We treated mouse with ESCC using monotherapy or combined therapy and analyzed the treated tumors. We found that targeting both the tumor bulk and Bmi1^+^ cells achieved the most efficacious tumor control. Consistently, a recent study on glioblastomas shows that combined Bmi1 targeting and another molecular targeting drug proved more effective than either agent alone both in culture and in vivo [32]. In addition, Bmi1 inhibitor PTC-209 has been proved to inhibit tumor growth by targeting CSC self-renewal in head and neck squamous cell carcinoma [18]. Our results demonstrate the significant efficacy of combined inhibition of Bmi1 and cisplatin, but we did not detect cures: the efficacy against each subtype was preferential, but not absolute. Future studies will determine how to achieve the desired results.

## 5. Conclusions

To sum up, our data provide specific experimental evidence that Bmi1^+^ cells are the cell origin of ESCC. Monotherapy alone against ESCC tumor growth near the limits of detection and combinational therapy of Bmi1 inhibitor and cisplatin were the most effective in reducing tumor burdens. Although the molecular basis of Bmi1-derived carcinogenesis and the clinical significance of Bmi1-derived ESCC have yet to be further studied, exploring the carcinogenic mechanism of multiple malignant tumors from the perspective of cell origin will provide us with new and promising therapeutic strategies.

## Data Availability

The data used to support the findings of this study are available from the corresponding author upon request.
